# Repeated moderate hypothermia leads to sustained glymphatic dysfunction and loss of vascular AQP4 polarization

**DOI:** 10.1186/s12987-026-00770-0

**Published:** 2026-02-16

**Authors:** Chenchen Liu, Na Liu, Nagesh C. Shanbhag, Marios Kritsilis, Nicholas Burdon Bèchet, Jari Jukkola, Ahmed M. Eltanahy, Roberta Battistella, Iben Lundgaard

**Affiliations:** 1 Department of Experimental Medical Science, Lund University, Lund, 223 62 Sweden; 2 Wallenberg Centre for Molecular Medicine, Lund University, Lund, Sweden; 3 Department of Neurology, Tongji Hospital, Tongji Medical College, Huazhong University of Science and Technology, Wuhan, China; 4 Egyptian Medical Board, Ministry of Health and Population, Giza, Egypt

**Keywords:** Hypothermia, Glymphatic system, Aquaporin-4

## Abstract

Cerebrospinal fluid (CSF) enters the brain along perivascular spaces in a manner that depends on aquaporin-4 (AQP4) polarization, facilitating brain clearance and is called the glymphatic system. While physiological factors such as cardiovascular activity and respiration are known to influence glymphatic function, the effects of hypothermia remain poorly understood. In this study, we used quantitative optical and light-sheet microscopy to track fluorescent CSF tracers in mice under ketamine–xylazine (KX) anesthesia at normothermia (37 °C), mild hypothermia (33 °C), and moderate hypothermia (30 °C). Acute mild hypothermia did not affect CSF influx, indicating tolerance to modest temperature fluctuations in KX anesthetized mice, whereas acute moderate hypothermia markedly impaired glymphatic transport by reducing tracer penetration and CSF outflow. Notably, repeated exposure to moderate hypothermia over four consecutive days induced a sustained suppression of glymphatic influx, which persisted even when assessed under normothermic conditions. This persistent dysfunction strongly correlated with the loss of perivascular AQP4 polarization. These findings demonstrate that repeated hypothermia exerts cumulative effects on glymphatic function, which may compromise brain waste clearance and contribute to cognitive impairment in vulnerable populations with impaired thermoregulatory capacity.

**Supplementary Information:**

The online version contains supplementary material available at 10.1186/s12987-026-00770-0.

## Introduction

Cerebrospinal fluid (CSF) plays an essential role in the central nervous system (CNS) by distributing nutrients and facilitating the clearance of metabolic waste. The glymphatic system is responsible for brain-wide CSF transport and mediation of CSF–interstitial fluid (ISF) exchange through perivascular space (PVS) flux, as demonstrated in both rodents and humans [1, 2]. The glymphatic system depends on the water channel aquaporin-4 (AQP4), which is localized to astrocytic endfeet and facilitates solute transport through the brain [3, 4]. Growing evidence suggests that the glymphatic system plays a critical role in clearing amyloid-β and preventing the progression of Alzheimer’s disease (AD) [5–8]. Previous studies on CSF transport have demonstrated that glymphatic-dependent CSF distribution is influenced by physiological parameters including heart rate, blood pressure, respiration, and anesthesia [9–14]. By contrast, much less is known about how body temperature affects glymphatic function. Decreases in core temperature can occur under physiological conditions, such as during natural sleep or following the induction of general anesthesia [15–17]. Previous experimental studies have shown that hypothermia, defined as a drop in core temperature below 35 °C [18], can exacerbate glymphatic dysfunction in the context of traumatic brain injury (TBI), but not in sham-operated mice [19]. However, in this study, glymphatic flow was not assessed during hypothermia itself, but only after rewarming, leaving the immediate effects of reduced temperature on the glymphatic system unresolved. Nevertheless, additional indirect evidence suggests that hypothermia may negatively affect the glymphatic system. Chronic exposure to mild hypothermia has been shown to promote amyloid-β accumulation in mice [20], and intraoperative hypothermia has been associated with a higher incidence of postoperative delirium and cognitive dysfunction [21, 22]. These findings raise the possibility that hypothermia may contribute to cognitive decline and dementia progression, potentially by impairing glymphatic clearance, although direct evidence is still lacking.

In this study, we hypothesize that hypothermia negatively affects CSF transport via the glymphatic system. To test this, we employed quantitative optical and light-sheet microscopy to track fluorescent CSF tracers in mice under ketamine/xylazine (KX) anesthesia at normothermia (37.0 °C), mild systemic hypothermia (33.0 °C), and moderate systemic hypothermia (30.0 °C), consistent with temperature ranges defined in previous experimental studies [23, 24]. Our results reveal that moderate hypothermia disrupts glymphatic transport into the PVS. Moreover, repeated moderate hypothermia exerts cumulative effects on glymphatic function, leading to a sustained impairment that is closely associated with loss of perivascular AQP4 polarization.

## Materials and methods

### Animals

Male C57BL/6 mice (aged 3–4 months, weight between 23 g and 30 g, Charles River Laboratories) were used for the experiment. Mice were housed at a stable temperature, with a 12/12 h dark/light cycle and ad libitum access to food and water. All experiments were approved by the Malmö-Lund Ethical Committee on Animal Research in Sweden (5.8.18–10258/18 and 5.8.18–20240/2021) and conducted according to the CODEX guidelines by the Swedish Research Council. This study complies with the ARRIVE (Animal Research: Reporting in Vivo Experiments) guidelines for reporting animal experiments.

### Hypothermia treatment and physiological parameters

Mice were anesthetized with a mixture of ketamine (100 mg/kg) and xylazine (20 mg/kg) intraperitoneally and then placed on a physiological monitoring system connected to a head fixation device for mice (Harvard Apparatus, catalog no. 75-1541, Holliston, USA). The surface temperature of the platform was set at 37 °C for normothermia (control), 33 °C for mild hypothermia, and 30 °C for moderate hypothermia [25]. As rectal and temporalis muscle temperature strongly correlate with brain temperature [26, 27] they were measured via a thermistor probe of the physiological monitoring system in the rectum and a 29G needle microprobe (Physitemp Instruments, Clifton, NJ) in the temporalis muscle. Typically, 10 min were required to achieve the target temperature. In the acute hypothermia cohort, mild and moderate hypothermia was maintained at 33 °C and 30 °C, respectively, for 60 min, followed by intracisternal CSF tracer infusion. In the repeated hypothermia cohort, the whole procedure lasted 2 h each time. These timepoints were motivated by previous studies which utilized 1-to-2-hour hypothermic regimens [25, 28, 29]. After 2 h, the mice fully recovered from the anesthesia at room temperature and then returned to the home cage. Mice underwent the same anesthesia and hypothermia procedure daily for four consecutive days, followed by intracisternal CSF tracer infusion under normothermic conditions on the fifth day.

Heart and respiration rates were recorded continuously throughout the experiment via the physiological monitoring system. Relative cerebral blood flow (CBF) of the cortex was measured by laser doppler flowmetry (Perimed, PeriFlux System 5000, Sweden). We exposed the right hemisphere skull and glued an optical filament (Perimed, catalog no. MT B500-0L240) on the skull close to the middle cerebral artery (MCA), then connected it to the probe (Perimed, catalog no. PROBE 418-1). The laser doppler value could be recorded once it was stable.

### Intracisternal CSF tracer infusion and in vivo imaging

Fluorescent CSF tracer (Bovine serum albumin conjugated to AlexaFluor647, BSA-647, 66 kDa, Invitrogen) was dissolved in artificial CSF (aCSF, in mM: 126 NaCl, 2.5 KCl, 1.25 NaH_2_PO_4_, 2 MgCl_2_, 2 CaCl_2_, 10 glucose, 26 NaHCO_3_, pH 7.4) at a concentration of 1% w/v. Anesthetized mice were fixed to the head fixation device (Harvard Apparatus, catalog no. 75-1541, Holliston, USA), and the cisterna magna (CM) was surgically exposed as described previously [30]. Briefly, a 30G needle connected to a 100µL Hamilton syringe through a polyethylene tubing (I.D. 0.28 mm, BD Intramedic) filled with the tracer was inserted into the CM. 10µL of 1% BSA-647 were injected at 1 µL /min for 10 min using a KDS Legato 100 single infusion syringe pump. For transcranial imaging, the skin was removed from the top of the head to expose the skull, and imaging was performed every minute from the beginning of intracisternal infusion using a Nikon SMZ25 stereo microscope at 0.5 × magnification (Plan Apo 0.5 × /0.078 NA). Tracer influx along the PVS of the MCA area was analyzed as the mean intensity images using an ROI of the MCA area on the in vivo time-lapse images. Following 30 min of tracer circulation, mice were decapitated. Brains were quickly removed and fixed overnight by immersion in 4% paraformaldehyde (PFA). For superficial cervical lymph nodes (SCLNs) in vivo imaging, upon completion of the tracer circulation, mice were placed supine under a Nikon SMZ25 stereo microscope with all the skin over the neck resected to reveal the SCLNs, and in vivo images of the SCLNs were taken at 0.5 × magnification (Plan Apo 0.5 × /0.078 NA).

### Ex vivo imaging of CSF tracer

The whole brains were imaged using a Nikon SMZ25 stereo microscope at 0.5 × magnification (Plan Apo 0.5 × /0.078 NA) and mean tracer intensities were analyzed using ImageJ. ROIs were defined as the dorsal and ventral cortex. Five coronal section slices (100 μm) spanning from + 2 to -2 mm relative to bregma were cut with a vibratome (Leica VT1200S) and imaged using a Nikon ECLIPSE Ti2 microscope at 4 × magnification (Plan Apo 4 × /0.2 NA). The mean tracer intensity of the whole slice was calculated using a Fiji macro developed by SciLifeLab Uppsala which uses the PerObjectEllipsefit plugin [31]. Brain subregions of interest, including dorsal cortex (DC), lateral cortex (LC), ventral cortex (VC), hippocampus (HIP), thalamus (THA), and hypothalamus (HT), were manually segmented and analyzed using ImageJ. PVS tracer penetration depth was measured by the distance from the cortical surface to where the tracer was no longer visible along PVS, using a Nikon Confocal A1RHD microscope at 20 × magnification (Plan Apo 20 × /0.75 NA).

Together with the brain, deep cervical lymph nodes (DCLNs), skull cap, and spinal cord were also isolated after tracer circulation and imaged using a Nikon SMZ25 stereo microscope at 0.5 × magnification (Plan Apo 0.5 × /0.078 NA). Dura was isolated from the skull cap and mounted. Tracer distribution in the dura was imaged by a Nikon ECLIPSE Ti2 microscope at 4 × magnification (Plan Apo 4 × /0.2 NA).

### Quantification of tracer levels in plasma and urine samples

After 30 min of tracer circulation, urine was directly drawn from the bladder using sterile needles and syringes, and blood samples were collected by cardiac puncture in heparinized tubes and then centrifuged at 2000×g for 15 min at 4 °C to separate plasma. BSA-647 level was quantified on a fluorescent plate reader (BMG Labtech CLARIOstar) with excitation and emission wavelengths set at 625 nm and 680 nm, respectively. The concentration of BSA-647 in plasma and urine was calculated from a standard curve.

### Optical tissue clearing and light-sheet imaging

After tracer circulation, mice were transcardially perfused with phosphate-buffered saline (PBS), followed by 4% PFA. Brains were extracted and fixed overnight by immersion in 4% PFA. The iDISCO+ (immunolabeling-enabled three-dimensional imaging of solvent-cleared organs) protocol was carried out as previously described [32, 33]. Briefly, tissues were dehydrated in increasing methanol/H_2_O series (20%, 40%, 60%, 80%, 100%, 100%, 1 h each), delipidated with methanol/dichloromethane (33%/66% for 3 h), and pure dichloromethane (2 times, 15 min each), and optically cleared with ethyl cinnamate for at least 1 week prior to imaging.

Following optical clearing, we performed light-sheet imaging of the samples using the LaVision-Miltenyi Biotec Blaze microscope. All samples were imaged using a 640 nm excitation wavelength and a 680/30 nm emission filter. The head-spine samples were imaged with the 1.1× objective (LaVision-Miltenyi Biotec MI PLAN ×1.1/0.1 NA, WD = 17 mm) in the transverse orientation at a step size of 5 μm. The mosaic acquisition setting (ImspectorPro64, LaVision Biotec) was used to obtain consecutive z-stacks with 10% overlap. Optically cleared brains were imaged as a single z-stack with the 1.1x objective and a step size of 2 μm.

### Light-sheet image analysis

The 16-bit TIFF images acquired from light-sheet imaging were imported to Arivis Vision4D v3.6.2 (Arivis AG) and converted to .sis files. Tiling scans of the head-spine samples were manually aligned in the XY axes based on visual inspection, using the Tile Sorter function of Arivis Vision4D. Representative 2D and 3D images and videos of the reconstructed samples were then acquired. Analysis of the 3D reconstructed brains was done using the built-in Analysis tools of Arivis Vision4D. The Intensity Threshold Segmenter tool was used for each brain image set (with uniform thresholding at a voxel intensity of 1024 out of 16536) and the sum voxel intensity and total volume of all voxels with an intensity value above the threshold were then automatically calculated by Arivis Vision4D as previously described [34].

We measured the mean tracer intensity in each of the transverse images of the brains in the z-stack in ImageJ to analyze the tracer distribution along the dorsoventral axis. Brains were also analyzed along the anteroposterior axis by dividing the images into five equal groups with the same ROIs [32]. PVS was also counted as previously described [35]. Briefly, orthogonal views of the inner cortical surface were generated to gain a cross-sectional view of the spaces. After reducing the background, we applied an intensity threshold, then converted to binary using a mask and counted the particles to generate the PVS number per mm^2^ and area coverage.

### Immunohistochemistry and analysis

Brain tissue after fixation were cut into 100 μm thick coronal sections using a Leica V1200S vibratome (Leica Biosystems). Brain sections at -2 mm from bregma were used for immunofluorescent staining. Slices were first permeabilized and blocked for 45 min in a solution of 0.5% Triton X-100, 5% normal donkey serum, and 1% BSA in PBS, and then incubated with primary antibodies (mouse anti-Glial Fibrillary Acidic Protein [GFAP], 1:500, Chemicon; rabbit anti-AQP4, 1:250, Merck Millipore). overnight at 4 °C, followed by three washes in 1xPBS and incubation with secondary antibodies (Donkey anti-mouse Alexa Fluor 488 and Donkey anti-rabbit Alexa Fluor 568, 1:1000, Invitrogen) for 2 h. Slices were then counterstained with 4′,6-diamidino-2-phenylindole (DAPI, 1:1000, Invitrogen) and/or tomato lectin (Lycopersicon esculentum, 1:200, Sigma Aldrich) for 30 min and then mounted. For evaluation of GFAP and AQP4 expression, images were acquired with a Nikon Confocal A1RHD microscope at 20 × magnification (Plan Apo 20 × /0.75 NA). Equivalent images of the dorsal cortex, lateral cortex, ventral cortex, hippocampus, thalamus, and hypothalamus were imaged for each slice (4 fields per region). To evaluate GFAP expressions, regions were uniformly thresholded and the area coverage of GFAP immunoreactivity (as a % of the whole area) was measured [36]. To measure AQP4 expression, mean AQP4 immunofluorescence intensity was measured in each region. The AQP4 polarization along vessels was evaluated as described previously [37, 38]. A 50 μm cross-sectional line was drawn using the line plot tool in ImageJ and centered on the blood vessel to include the AQP4 signal from the vascular endfeet and the surrounding parenchyma. The polarization index was calculated as the average peak value in the vascular endfeet divided by average parenchyma fluorescence intensity. Six vessels were analyzed per region. For each subregion, the normalized GFAP expression, AQP4 expression, and AQP4 polarization values were normalized to the highest value for ease of visualization and comparison, ranging from 0 to 1. All data were evaluated by an experimenter blinded to group allocation.

### Statistics

All statistical analysis was performed in GraphPad Prism version 9.0, Normality was checked by the Shapiro-Wilk test. An unpaired t-test was used for pairwise comparisons. For more than two groups, one-way ANOVA analysis with Tukey’s multiple comparisons tests was carried out. In cases of a non-normal distribution, a nonparametric Mann–Whitney test was used for comparisons between two groups, and a nonparametric Kruskal–Wallis test was used for comparisons between more than two groups. For two independent variables, two-way ANOVA with Tukey’s multiple comparisons tests was used. Regression analysis assessed simple correlations using Pearson’s correlation coefficient. *p*-values < 0.05 were deemed significant. Boxplots in all figures indicate minimum, first quartile, median, third quartile, and maximum values. Individual animals are shown as colored dots. In the line graphs, thick lines show group means, and shaded regions indicate standard error of the mean (SEM).

## Results

### Acute moderate hypothermia impairs glymphatic function

**Fig. 1:**
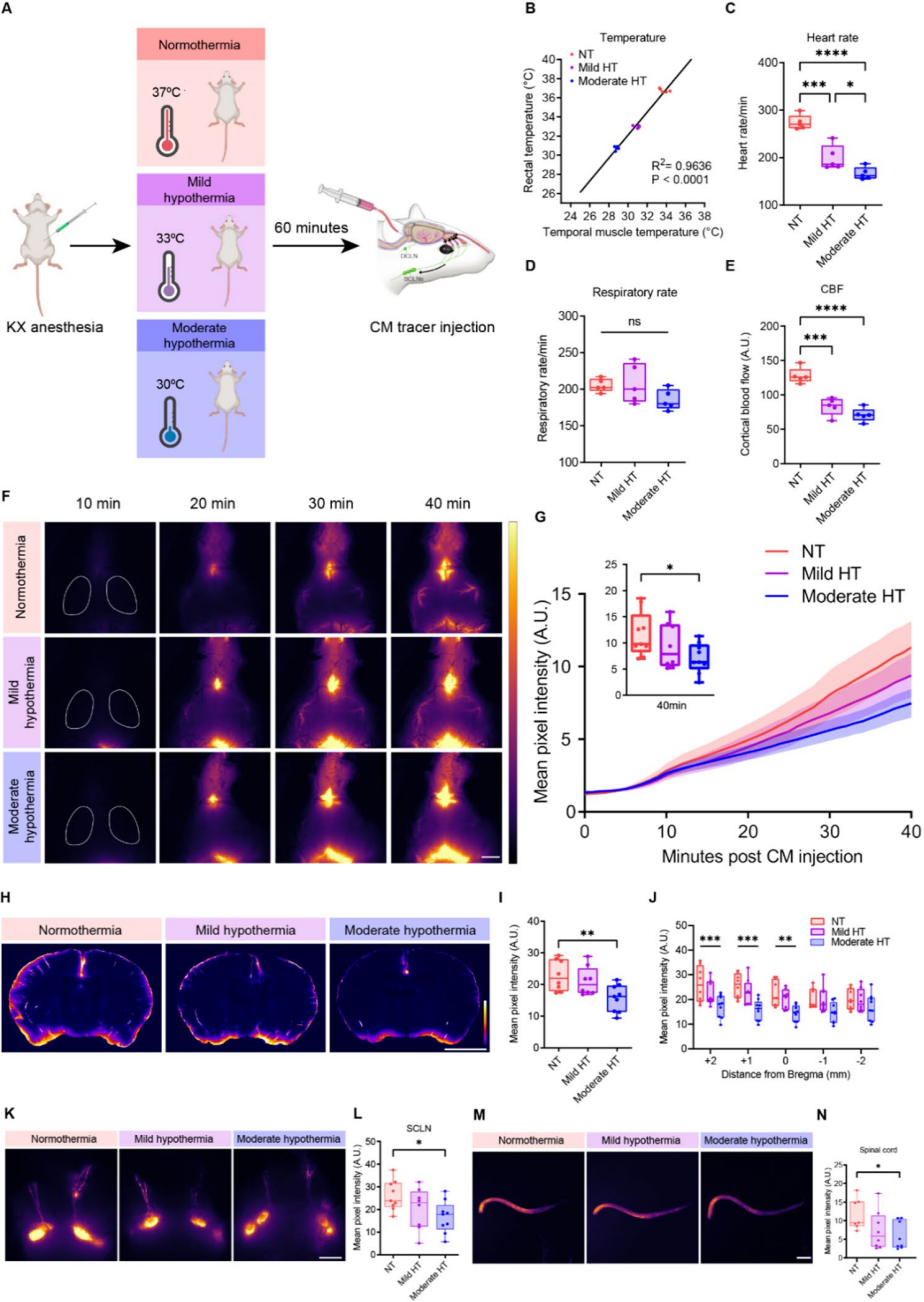
Acute moderate hypothermia impairs glymphatic function. (**A**) Experimental schematic. (**B**) Rectal and temporalis muscle temperatures measured during tracer circulation. Linear regression between temporalis muscle and rectal temperature (R² = 0.9636, p < 0.0001; n = 5 per group). (**C-D**) Heart rate and respiratory rate recorded during tracer circulation (one-way ANOVA with Tukey’s multiple comparisons test, n = 5 per group). (**E**) Relative CBF measured by laser Doppler flowmetry during tracer circulation (one-way ANOVA with Tukey’s multiple comparisons test, n = 5 per group). (**F**) Representative transcranial in vivo recordings of CSF tracer influx following CM injection. The white line outlines the middle cerebral artery region. Scale bar, 2.5 mm. (**G**) Quantification of tracer mean pixel intensity following CM injection. The inset displays fluorescence intensity at 40 min with boxplots (one-way ANOVA with Tukey’s multiple comparisons test, n = 8–9 per group). (**H**) Representative coronal brain sections. Scale bar, 2.5 mm. (**I**) Quantification of tracer influx across coronal brain slices, averaged from five slices per brain (one-way ANOVA with Tukey’s multiple comparisons test, n = 8–9 per group). (**J**) Quantification of tracer influx in coronal brain slices by location (–2, − 1, 0, + 1, +2 mm from bregma) (two-way ANOVA with Tukey’s multiple comparisons test, n = 8–9 per group). (**K**–**L**) Representative in vivo images and quantification of tracer distribution in the SCLNs. Scale bar, 2.5 mm (one-way ANOVA with Tukey’s multiple comparisons test, n = 8–9 per group). (**M-N**) Representative ex vivo images and quantification of tracer distribution in the spinal cord. Scale bar, 5 mm (one-way ANOVA with Tukey’s multiple comparisons test, n = 7–8 per group). ns, not significant; *p < 0.05; **p < 0.01; ***p < 0.001; ****p < 0.0001. NT, normothermia; HT, hypothermia; CBF, cerebral blood flow; SCLNs, superficial cervical lymph nodes

To investigate the effects of hypothermia, body temperature was controlled under KX anesthesia and evaluated by rectal and temporalis muscle measurements (Fig. 1A). Temporalis muscle temperature was consistently lower than rectal temperature, however, the two measures were strongly correlated, suggesting a cooling of the head during systemic hypothermia (*R²* = 0.9636, *p* < 0.0001; Fig. 1B). Since glymphatic function is influenced by the cardiac and respiratory cycles [11], we closely monitored physiological parameters including heart rate, respiratory rate, and relative CBF during acute hypothermia. The heart rate was significantly reduced during acute hypothermia (Fig. 1C). Although there was a trend toward a reduced respiratory rate, no significant difference was detected (Fig. 1D). Relative CBF decreased during both mild and moderate hypothermia compared with normothermia (Fig. 1E).

To assess glymphatic influx, we injected a fluorescein-conjugated protein-based tracer (BSA-AlexaFluor647) into the cisterna magna (CM) and allowed the tracer to circulate for 30 min. In vivo transcranial imaging revealed decreased CSF tracer influx into the brain in the MCA region during moderate hypothermia compared with normothermia, measured 30 min after injection (Fig. 1F–G). After imaging, brains were collected and immersion-fixed in 4% PFA. CSF tracer distribution was also quantified using ex vivo 100-µm coronal sections (Fig. 1H). Consistent with the in vivo results, the moderate hypothermia group exhibited significantly reduced parenchymal tracer influx compared with normothermia (Fig. 1I–J). More detailed quantification revealed that perivascular tracer penetration depth in the moderate hypothermia group was significantly lower than in both the normothermia and mild hypothermia groups (Supplementary Fig. 1). Taken together, these data demonstrate that acute moderate hypothermia decreased tracer influx along the MCA and reduced CSF penetration into the brain parenchyma.

We next asked whether CSF outflow pathways were altered during acute hypothermia. To address this, we compared in vivo tracer filling of the SCLNs 30 min after injection of the tracer in the CM. Quantification revealed a significant reduction in tracer intensity in the moderate hypothermia group compared with normothermia (Fig. 1K–L). Ex vivo fluorescence imaging further showed that tracer signal in the spinal cord was also reduced by half in moderate hypothermia compared with normothermia (Fig. 1M–N). By contrast, no significant differences were observed in tracer distribution to the dura, skull cap, or the DCLNs (Supplementary Fig. 2A–F). Finally, to test whether CSF outflow into blood and urine was affected, plasma and urine were collected after 40 min of tracer circulation. Tracer concentrations in plasma and urine did not differ significantly among the three groups (Supplementary Fig. 2G–H). Together, these results suggest that in parallel with reduced CSF influx into the brain, CSF outflow pathways to the SCLNs and spinal cord were also impaired under acute moderate hypothermic conditions.

### Light-sheet imaging confirmed global impairments in glymphatic function during acute moderate hypothermia

**Fig. 2:**
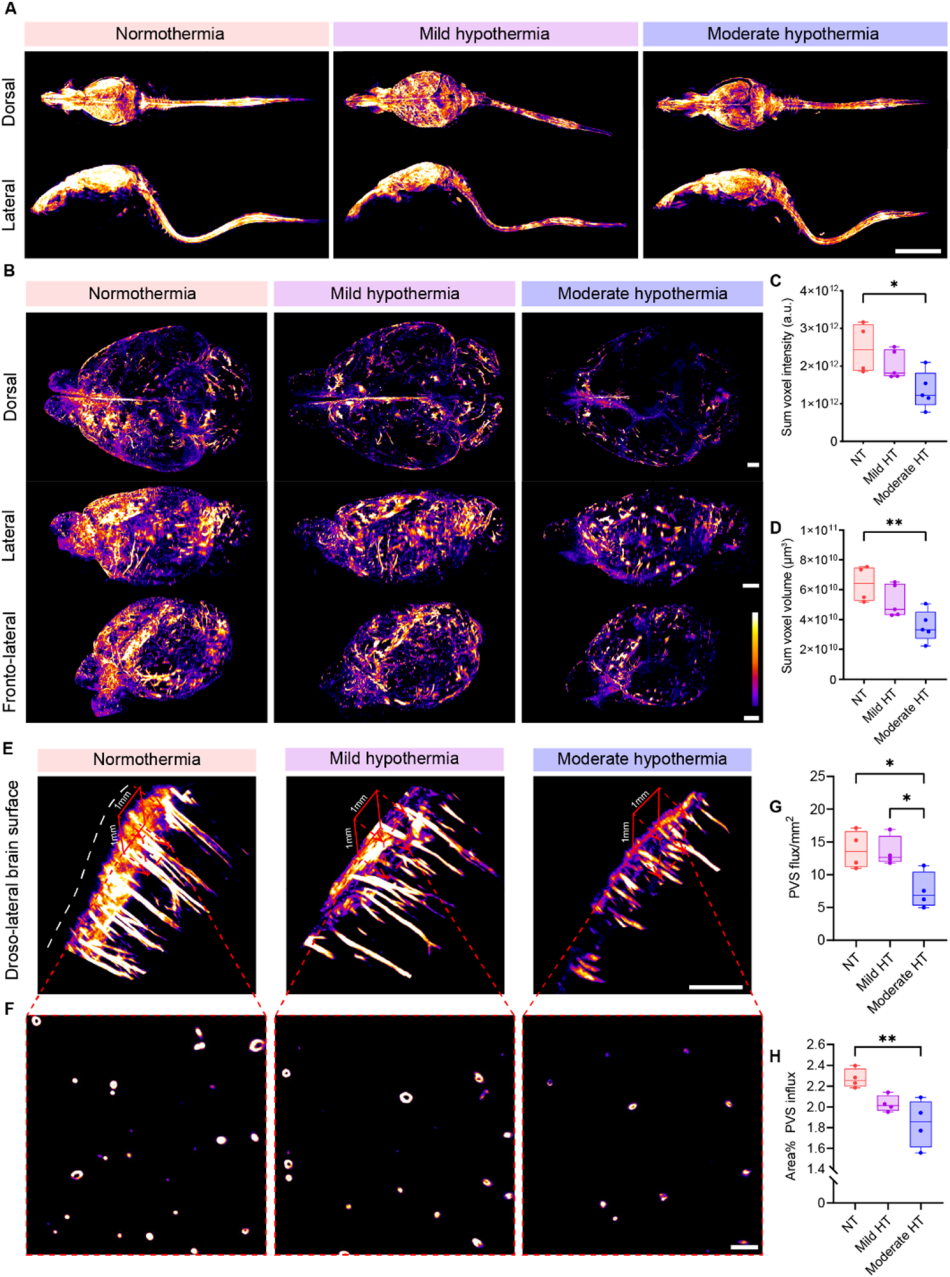
Light-sheet imaging reveals global impairments in glymphatic function during acute moderate hypothermia.** (A)** Representative light-sheet images of dorsal and lateral views of 3D tracer reconstruction in the cleared whole head, lymph nodes, and spinal column under normothermia (red), mild hypothermia (purple), and moderate hypothermia (blue). Scale bar, 10 mm. **(B)** Representative light-sheet images of dorsal, lateral, and fronto-lateral views of 3D tracer reconstruction in the cleared brain from all three groups. Scale bar, 2 mm. **(C)** Quantification of summed tracer intensity in the cleared brain (one-way ANOVA with Tukey’s multiple comparisons test, *n* = 4–5 per group). **(D)** Quantification of summed tracer volume in the cleared brain (one-way ANOVA with Tukey’s multiple comparisons test, *n* = 4–5 per group). **(E)** 3D reconstructions from light-sheet imaging of the dorso-lateral cortical surface showing PVS tracer penetration. A 1 × 1 mm² ROI was used to count perivascular influx sites projecting into the brain. Scale bar, 500 μm. **(F)** Representative image of a cross-section through perivascular channels parallel to the cortical surface. Scale bar, 100 μm. **(G)** Quantification of perivascular influx sites per mm² of cortex (one-way ANOVA with Tukey’s multiple comparisons test, *n* = 4 per group). **(H)** Quantification of cortical surface area covered by perivascular influx (one-way ANOVA with Tukey’s multiple comparisons test, *n* = 4 per group). *p < 0.05; **p < 0.01. NT, normothermia; HT, hypothermia; PVS, perivascular space; ROI, region of interest

In vivo optical imaging and brain slice analysis provide valuable insights into glymphatic function but are limited in field of view and spatial resolution. To overcome these limitations, we performed light-sheet imaging of optically cleared whole brains to assess hypothermia-induced glymphatic disturbances in greater detail. 3D reconstructions of the whole head, along with lymph nodes and the spinal column, were generated for qualitative assessment (Fig. 2A), and quantitative analyses were performed on whole-brain samples (Fig. 2B; Supplementary Videos 1–3). The summed tracer intensity was reduced by almost half in moderate hypothermia compared with normothermia (Fig. 2C). A similar reduction was observed in the volume of tracer coverage (Fig. 2D). More detailed quantification showed that under acute moderate hypothermia, the reduction in tracer influx occurred significantly in the central regions of the brain along both the anteroposterior and dorsoventral axes (Supplementary Fig. 3). This indicates that in the moderate hypothermia group, tracer penetration into deeper brain regions was restricted. Consistent with the slice-based quantifications, light-sheet imaging further confirmed reduced tracer influx into the PVS under moderate hypothermia (Fig. 2E–H). Taken together, the light-sheet data demonstrates global impairments in glymphatic function during acute moderate hypothermia.

### Sustained reduction of glymphatic influx after repeated moderate hypothermia

Given that acute moderate hypothermia markedly reduced CSF influx, we next asked whether repeated hypothermic episodes would have cumulative or sustained effects on glymphatic function, even after returning to normal temperature. To address this, we employed a paradigm of repeated KX-anesthesia–induced hypothermia, followed by experiments performed under normothermic conditions after a day of recovery. To rule out the possibility that repeated anesthesia alone affected glymphatic function, we first compared CSF dynamics between control mice and those subjected to repeated KX anesthesia under normothermic conditions (Supplementary Fig. 4A). In vivo transcranial imaging revealed no significant differences in CSF tracer influx between groups in the MCA region, cortex, olfactory bulb, or olfactory cistern region (Supplementary Fig. 4B–F). Consistent results were obtained from the analysis of tracer distribution in coronal brain sections (Supplementary Fig. 4G-I). Together, these data demonstrate that repeated KX anesthesia itself did not affect glymphatic function.

**Fig. 3:**
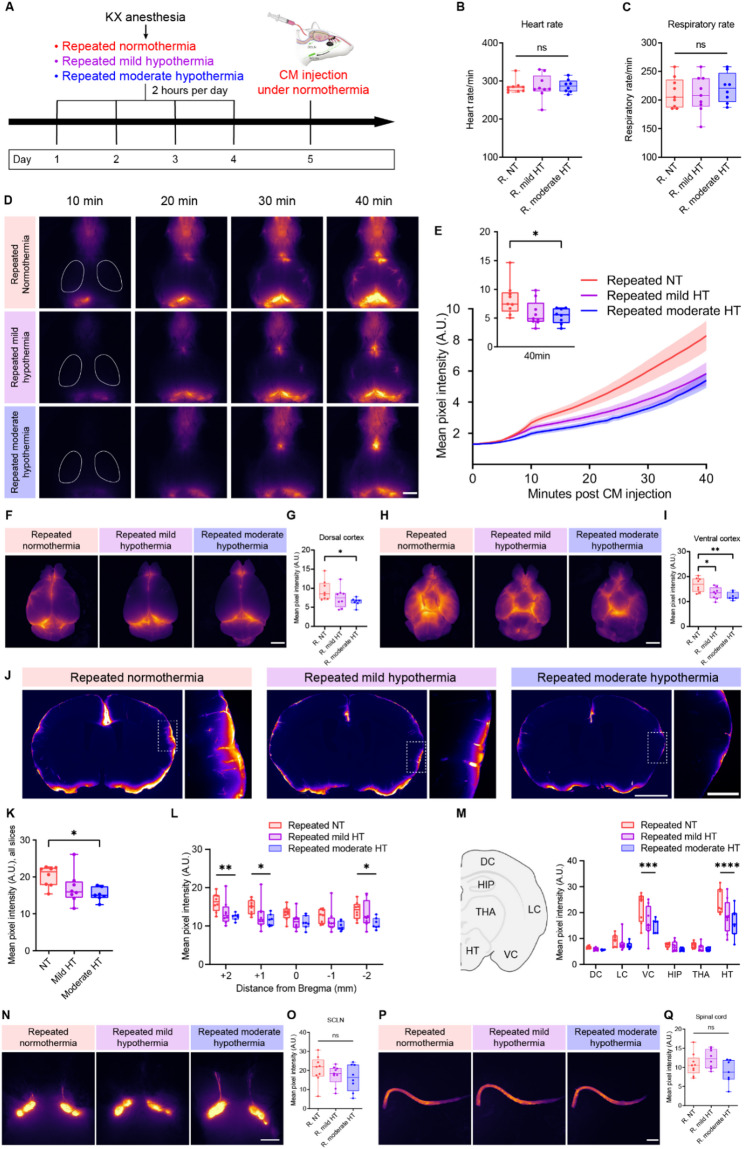
Repeated moderate hypothermia impairs glymphatic influx.** (A)** Experimental schematic. **(B-C)** Heart rate and respiratory rate recorded during tracer circulation (one-way ANOVA with Tukey’s multiple comparisons test, *n* = 8–9 per group). **(D-E)** Representative in vivo transcranial images and quantification of CSF tracer influx after cisterna magna injection. The white line outlines the middle cerebral artery region. Scale bar, 2.5 mm. Inset shows tracer intensity at 40 min with boxplots (one-way ANOVA with Tukey’s multiple comparisons test, *n* = 8–9 per group). **(F–I)** Representative ex vivo dorsal and ventral brain surface images and quantification of tracer distribution. Scale bar, 2.5 mm (one-way ANOVA, Tukey’s test; *n* = 7–8). **(J)** Representative coronal brain sections. Scale bar, 2.5 mm, 500 μm. **(K)** Quantification of tracer influx across coronal brain slices, averaged from five slices per brain (one-way ANOVA with Tukey’s multiple comparisons test, *n* = 7–8 per group). **(L)** Quantification of tracer influx in coronal brain slices by location (–2, − 1, 0, + 1, +2 mm from bregma) (two-way ANOVA with Tukey’s multiple comparisons test, *n* = 7–8 per group). **(M)** Quantification of tracer influx in coronal brain slices by brain region (two-way ANOVA with Tukey’s multiple comparisons test, *n* = 7–8 per group). **(N–O)** Representative in vivo images and quantification of tracer distribution in the SCLNs. Scale bar, 2.5 mm (one-way ANOVA with Tukey’s multiple comparisons test, *n* = 8–9 per group). **(P–Q)** Representative images and quantification of tracer distribution in the spinal cord. Scale bar, 5 mm (one-way ANOVA with Tukey’s multiple comparisons test, *n* = 7–9 per group). ns, not significant; *p < 0.05; **p < 0.01; ***p < 0.001; ****p < 0.0001. R, repeated; NT, normothermia; HT, hypothermia; DC, dorsal cortex; LC, lateral cortex; VC, ventral cortex; HIP, hippocampus; THA, thalamus; HT, hypothalamus

We then carried out a repeated hypothermia experiment where animals were subjected to four consecutive days of induced normothermia, mild hypothermia, or moderate hypothermia under KX anesthesia for 2 h (Fig. 3A). On the fifth day, glymphatic function was evaluated by CM injection of a fluorescent tracer at normothermia. Heart rate and respiratory rate were also measured under normothermia, and no significant differences were observed among groups (Fig. 3B–C). Notably, CSF tracer influx into the brain along the MCA region was reduced at 30 min of tracer circulation time in the repeated moderate hypothermia group at normothermia compared with repeated normothermia (Fig. 3D–E). Ex vivo brain imaging further showed that tracer intensity at both the dorsal and ventral cortical surfaces was significantly decreased at normothermia in repeated moderate hypothermia compared with repeated normothermia (Fig. 3F–I), consistent with slice-based quantification (Fig. 3J–M). By contrast, tracer intensity in the SCLNs and spinal cord did not differ significantly among groups (Fig. 3N–Q). Together, these findings demonstrate that repeated moderate hypothermia impaired CSF influx into the brain, and that this impairment persisted at least one day after animals returned to normothermia.

### Reduced perivascular AQP4 polarization is associated with CSF influx diminishment in repeated moderate hypothermia

**Fig. 4:**
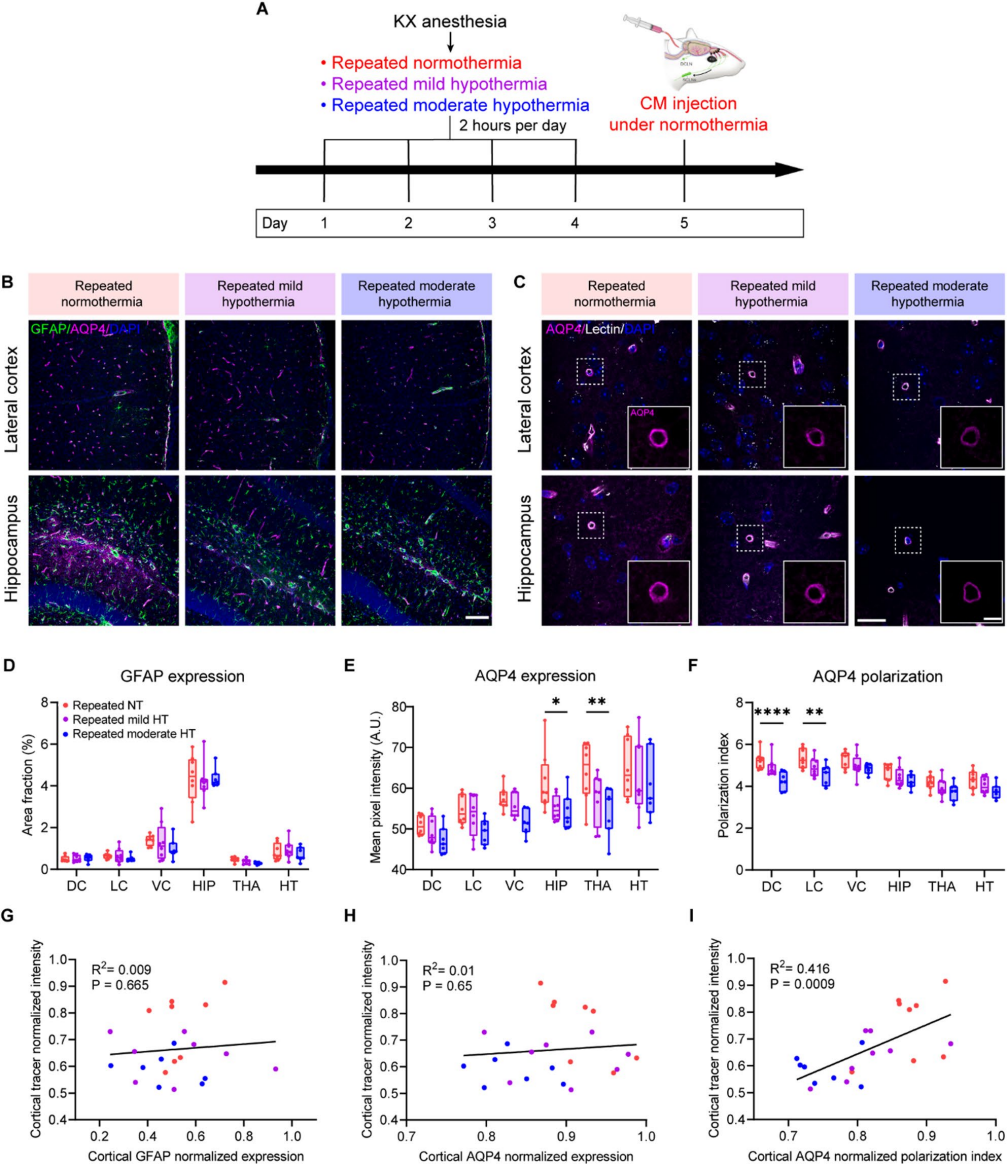
Reduction of perivascular AQP4 polarization is associated with CSF influx impairment in repeated hypothermia.** (A)** Experimental schematic. **(B)** Representative confocal images showing immunostaining of GFAP (green) and AQP4 (magenta) in the lateral cortex and hippocampus. Scale bar, 100 μm. **(C)** Representative confocal images of AQP4 (magenta) staining around lectin (white)-labelled blood vessels in the lateral cortex and hippocampus. Scale bar, 20 μm; inset, 5 μm. **(D)** Quantitative analysis of GFAP area fraction by region (two-way ANOVA with Tukey’s multiple comparisons test, *n* = 7–8 per group). **(E)** Quantitative analysis of global AQP4 mean fluorescent intensity by region (two-way ANOVA with Tukey’s multiple comparisons test, *n* = 7–8 per group). **(F)** Quantitative analysis of the AQP4 polarization index by region (two-way ANOVA with Tukey’s multiple comparisons test, *n* = 7–8 per group). **(G)** Linear regression analysis showed that GFAP expression was not significantly associated with tracer penetration in the cortex (*n* = 7–8 per group). All values were normalized to the highest value for ease of visualization and comparison. **(H)** Global AQP4 mean fluorescent intensity was not significantly associated with tracer penetration in the cortex (*n* = 7–8 per group). **(I)** AQP4 polarization was positively correlated with tracer penetration in the cortex (*n* = 7–8 per group). ns, not significant; *p < 0.05; **p < 0.01; ****p < 0.0001. NT, normothermia; HT, hypothermia; DC, dorsal cortex; LC, lateral cortex; VC, ventral cortex; HIP, hippocampus; THA, thalamus; HT, hypothalamus; GFAP, glial fibrillary acidic protein; AQP4, aquaporin-4

Since glymphatic influx and clearance is critically regulated by the polarized distribution of AQP4 channels at astrocytic endfeet [39], we next assessed astrocytic responses—particularly changes in AQP4 polarization—after repeated hypothermia using immunostaining (Fig. 4A-C). Whereas GFAP expression remained unchanged across subregions (Fig. 4D), AQP4 expression was markedly reduced in the repeated moderate hypothermia group compared with normothermia in the hippocampus and thalamus (Fig. 4E). More importantly, repeated moderate hypothermia led to a trend in reduction in AQP4 polarization across all evaluated brain regions, with significant decreases observed in both the dorsal and lateral cortex (Fig. 4F). This loss of AQP4 polarization was not observed in the acute hypothermia cohort, except in the ventral cortex (Supplementary Fig. 5). We hypothesized that reduced perivascular AQP4 polarization after repeated hypothermia hampers glymphatic influx. Correlation analysis confirmed that lower cortical AQP4 polarization was associated with reduced cortical tracer intensity (Fig. 4I). By contrast, no significant associations were found between cortical tracer intensity and either GFAP expression (Fig. 4G) or overall AQP4 expression levels (Fig. 4H). Beyond the cortex, perivascular AQP4 polarization was also positively correlated with CSF influx in the thalamus and hypothalamus, but not in the hippocampus (Supplementary Fig. 6). Overall, these findings demonstrate that reduced AQP4 polarization after repeated hypothermia was linked to impaired glymphatic function, identifying astrocytic endfeet as a key vulnerability to repeated hypothermic exposure.

## Discussion

In this study, we demonstrated that glymphatic function is impaired during acute moderate hypothermia, as indicated by reduced tracer penetration through PVS and diminished CSF outflow to the spinal cord and the SCLNs. Notably, the most striking finding emerged from the repeated hypothermia experiments. Here, glymphatic function was assessed after the mice had returned to normothermia, yet glymphatic influx remained suppressed and was accompanied by a loss of perivascular AQP4 polarization. These results suggest that repeated hypothermia may not simply exert an acute, temperature-dependent effect, but could instead induce a more sustained dysfunction of glymphatic transport.

Our results showed that glymphatic function remained intact under acute mild hypothermia (33.0 °C), indicating that the system can tolerate temperature fluctuations to some degree. This thermostability may be physiologically relevant during sleep, when body temperature typically decreases, and is also consistent with the safety of most therapeutic hypothermia protocols, which are generally conducted at ~ 33.0 °C [40–42]. In contrast, acute moderate hypothermia (30.0 °C) impaired glymphatic function, which was accompanied by reductions in heart rate and CBF, suggesting that the impairment largely reflects a systemic slowing of metabolism and circulation [43–45].

By including a control group subjected to repeated KX anesthesia alone, our study demonstrated that repeated KX anesthesia by itself did not compromise glymphatic function; the impairment emerged only in the presence of moderate hypothermia. We further showed that this persistent glymphatic dysfunction correlated with decreased perivascular AQP4 polarization following repeated moderate hypothermia. Previous studies have reported that hypothermia attenuates pathological global AQP4 upregulation in disease models of ischemia [46, 47], intracerebral hemorrhage [48], TBI [49], and cardiac arrest [50]—an effect generally considered neuroprotective, as excessive AQP4 contributes to brain edema under pathological conditions [51]. However, our findings reveal that repeated moderate hypothermia further reduces AQP4 polarization even in the absence of pathology. While transient hypothermia may be beneficial in acute injury settings by limiting pathological AQP4 upregulation, repeated exposure appears to compromise normal glymphatic physiology by disrupting AQP4 localization at astrocytic endfeet—highlighting a potential double-edged effect of hypothermia on brain fluid regulation. Although moderate therapeutic hypothermia (32–33.9 °C) [41, 52, 53] is typically applied within a temperature range comparable to the mild hypothermia used in this study, the potential effects of prolonged hypothermia remain unclear and should be addressed in future studies.

The mechanism by which hypothermia—especially repeated hypothermia—affects AQP4 polarization remains largely unclear, but several potential pathways can be considered. First, decreased AQP4 polarization might partly result from a reduction in overall AQP4 expression in astrocytes under lowered temperature. Both our data under physiological conditions and previous reports in disease models [46–50] have shown reduced global AQP4 after hypothermia. However, in vitro studies on this aspect have yielded divergent results. For example, Lo Pizzo et al. reported decreased AQP4 expression in cultured astrocytes following hypothermia [54], whereas Salman et al. observed no change in total protein levels in cortical astrocytic cultures [55]. Second, hypothermia may directly alter AQP4 polarization at astrocytic endfeet. Salman et al. reported increased AQP4 membrane localization under hypothermia via a TRPV4 (transient receptor potential vanilloid 4)/calmodulin-mediated mechanism [55]. This supports the notion that AQP4 cell membrane anchoring is temperature sensitive and hints at potential differences between astrocytic responses to hypothermia in vitro and in vivo. Meanwhile, some indirect in vivo evidence has also indicated that hypothermia can reduce AQP4 polarization through intracellular signaling pathways. Inhibition of the extracellular signal-regulated kinase (ERK) pathway was shown to rescue AQP4 depolarization by preventing β-dystroglycan cleavage in an ischemia model [56], whereas ERK signaling is typically activated under hypothermic conditions [57, 58]. These findings raise the possibility that ERK activity might serve as a mechanistic link between hypothermia and AQP4 depolarization, although further in vivo studies are required to confirm this relationship.

Our findings may be particularly relevant to individuals with diminished thermoregulatory capacity, such as neonates, patients with AD, and the elderly, since their susceptibility to repeated short episodes of hypothermia is considerably higher under physiological or clinical challenges. In older adults, thermoregulatory function declines due to impaired cold perception, reduced metabolic heat production, and slower vasoconstrictive responses [59, 60]. Reflecting these vulnerabilities, intraoperative hypothermia is more frequent and severe in elderly patients compared with younger adults [59, 61]. Neonates are similarly at high risk, with intraoperative hypothermia reported in 54–79% of cases, largely because their thermoregulatory system is immature [62, 63]. Thermoregulatory dysfunction has also been observed in AD patients [64], and preclinical experiments further confirmed that AD mice are more vulnerable to cold exposure compared with age-matched controls [65, 66] Hypothermia has been implicated as a risk factor for cognitive decline, with tau hyperphosphorylation induced by hypothermia proposed as one potential mechanism [67]. Our study identifies an additional, glymphatic-related mechanism: repeated hypothermia episodes may progressively impair AQP4-dependent glymphatic clearance, contributing to long-term neurological dysfunction [68–71]. These mechanistic insights might help explain the previously reported higher incidence of postoperative cognitive dysfunction and delirium following general anesthesia in elderly patients compared to younger adults [72, 73], further highlighting the importance of careful thermal management in these vulnerable populations.

This study has several limitations. Approximately 30-min circulation period following CM injection is the standard termination point within the glymphatic field [1, 74, 75], and while we were mostly interested in capturing this influx-dominant phase, this experiment might not capture the full spectrum of effects hypothermia might have on tracer kinetics.

Another potential limitation of this study is related to key physiological differences in thermoregulatory strategies between mice and human, as mice regularly utilize torpor as a survival mechanism [76, 77], whereas human physiology does not allow reaching this state of low energy expenditure. To standardize the hypothermic regimen for our proof-of-concept study, only young adult male mice were used due to the cyclical hormonal changes which females have that could interfere with core temperature [78] and cause complications in executing comparable hypothermic regimens between sexes.

As our current study establishes moderate hypothermia as a model for causing brain-wide glymphatic disruption, future studies should investigate the direct cellular effects of hypothermia in cerebral cell populations, particularly in astrocytes. Mice lacking AQP4 water channels have been classically used in the field to study chronic disruption of glymphatic activity [1]. Therefore, it would be relevant to compare AQP4 knockout mice with wild-type mice which have undergone repeated moderate hypothermic regimens to investigate whether the decrease in glymphatic influx is mediated through AQP4 alone. Combining AQP4 mice with moderate hypothermic regimens could represent a profoundly disrupted glymphatic model, perhaps enabling more precise investigations in alternative models for rescuing glymphatic function.

## Supplementary Information

Below is the link to the electronic supplementary material.

Supplementary Material 1: Representative 3D light-sheet reconstruction of tracer distribution in a cleared brain from the normothermia group. Scale bar: 1 mm.

Supplementary Material 2: Representative 3D light-sheet reconstruction of tracer distribution in a cleared brain from the mild hypothermia group. Scale bar: 1 mm.

Supplementary Material 3: Representative 3D light-sheet reconstruction of tracer distribution in a cleared brain from the moderate hypothermia group. Scale bar: 1 mm.

Supplementary Material 4

## Data Availability

The data of this study are available on request from the corresponding author.

## Abbreviations

AD: Alzheimer’s disease
AQP4: Aquaporin–4
CBF: Cerebral blood flow
CNS: Central nervous system
CM: Cisterna magna
CSF: Cerebrospinal fluid
DC: Dorsal cortex
DCLNs: Deep cervical lymph nodes
GFAP: Glial fibrillary acidic protein
HIP: Hippocampus
HT: Hypothalamus
ISF: Interstitial fluid
iDISCO+: Immunolabeling–enabled three–dimensional imaging of solvent–cleared organs
KX: Ketamine/xylazine
LC: Lateral cortex
MCA: Middle cerebral artery
PVS: Perivascular space
SCLNs: Superficial cervical lymph nodes
TBI: Traumatic brain injury
THA: Thalamus
VC: Ventral cortex
