# Supplementary material for: Repeated moderate hypothermia leads to sustained glymphatic dysfunction and loss of vascular AQP4 polarization
Source: Fluids Barriers CNS. 2026 Feb 16;23:48. doi: 10.1186/s12987-026-00770-0 (PMC13023170; doi:10.1186/s12987-026-00770-0)
Supplement: Supplementary file 4 — Supplementary Material 4 [file 12987_2026_770_MOESM4_ESM.docx]

**Supplementary material**

**Supplementary Figures**

**
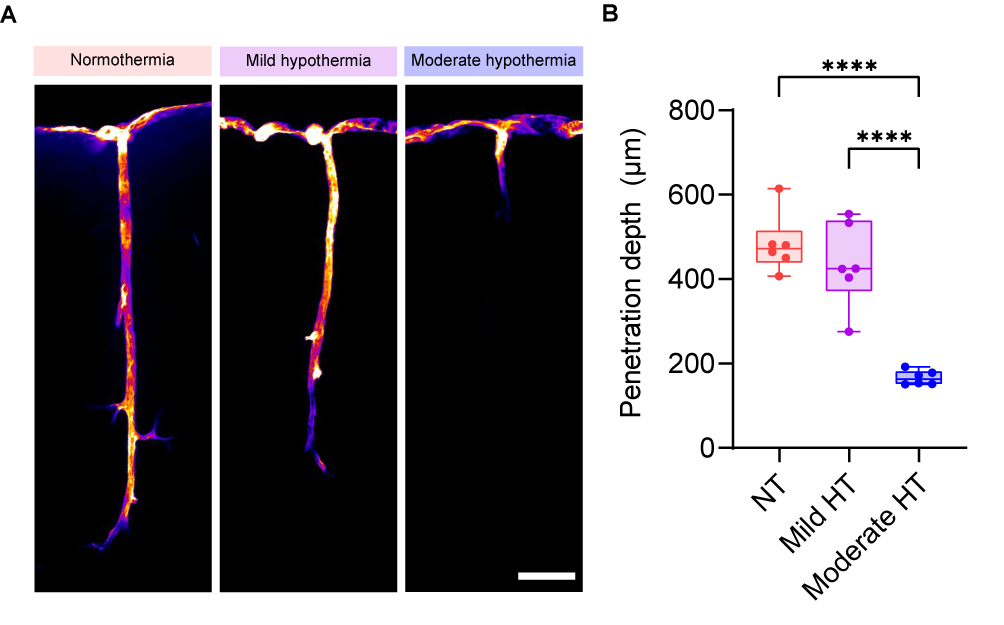
Supplementary Figure 1. Reduced perivascular tracer penetration during acute moderate hypothermia.** **(A)** Representative confocal images showing perivascular tracer penetration depth from the brain surface under normothermia (red), mild hypothermia (purple), and moderate hypothermia (blue). Scale bar, 100 µm. **(B)** Quantitative analysis of perivascular tracer penetration depth across the three groups (one-way ANOVA with Tukey’s multiple comparisons test, *n* = 6 per group, 10 vessels per animal). *****p* < 0.0001. NT, normothermia; HT, hypothermia.

**
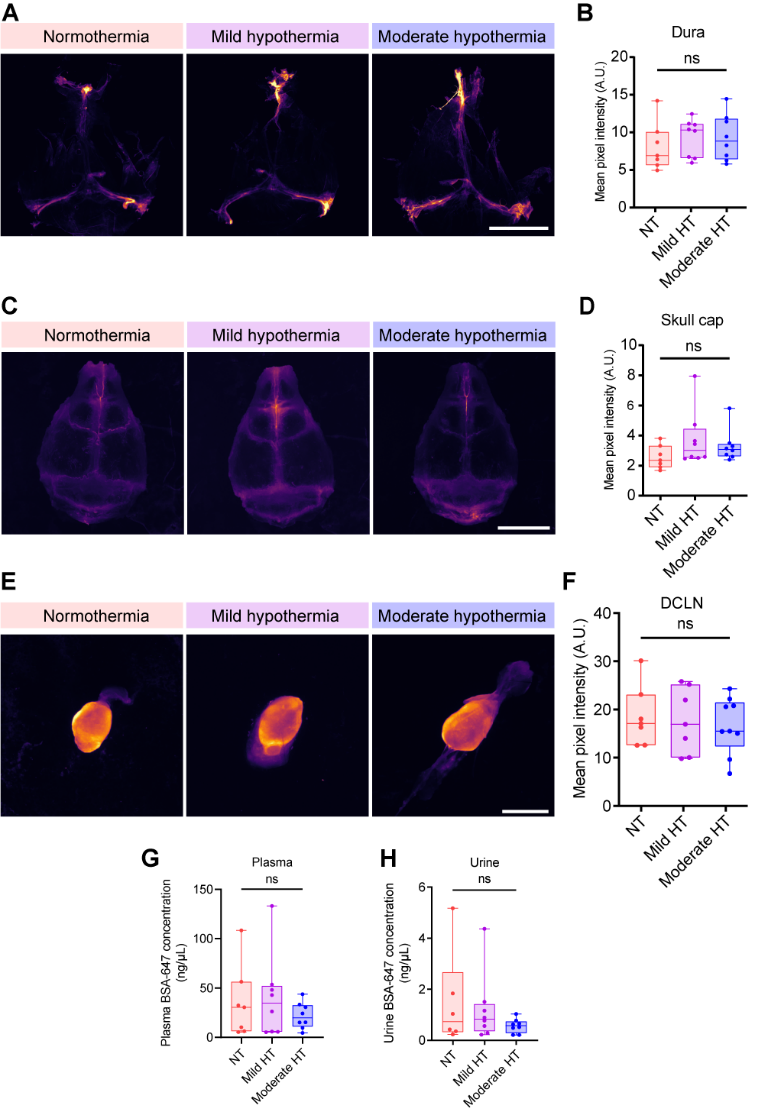
Supplementary Figure 2. Tracer distribution in CSF outflow pathways during acute hypothermia. (A–B)** Representative images and quantification of tracer distribution in the dura. Scale bar, 5 mm (one-way ANOVA with Tukey’s multiple comparisons test, *n* = 7–8 per group). **(C–D)** Representative images and quantification of tracer distribution in the skull cap. Scale bar, 5 mm (one-way ANOVA with Tukey’s multiple comparisons test, *n* = 7–8 per group). **(E–F)** Representative images and quantification of tracer distribution in the DCLN. Scale bar, 5 mm (one-way ANOVA with Tukey’s multiple comparisons test, *n* = 7–8 per group). **(G)** Quantitative analysis of tracer concentration in plasma (one-way ANOVA with Tukey’s multiple comparisons test, *n* = 7–8 per group). **(H)** Quantitative analysis of tracer concentration in urine (one-way ANOVA with Tukey’s multiple comparisons test, *n* = 6–8 per group). ns, not significant. NT, normothermia; HT, hypothermia; DCLN, deep cervical lymph node.


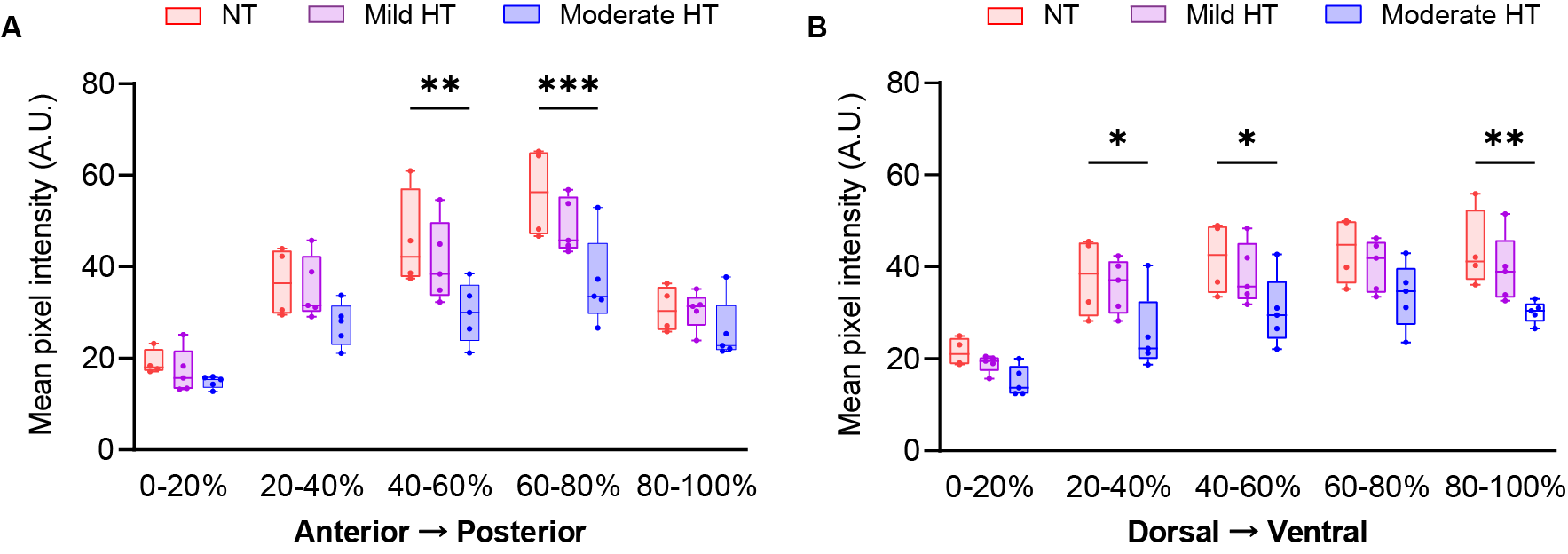
**Supplementary Figure 3. Reduced tracer penetration into deeper brain regions during moderate hypothermia by light-sheet. (A)** Quantitative analysis of mean tracer intensity in five 20-percentile regions across the anteroposterior axis (two-way ANOVA with Tukey’s multiple comparisons test, *n* = 4–5 per group). **(B)** Quantitative analysis of mean tracer intensity in five 20-percentile regions across the dorsoventral axis (two-way ANOVA with Tukey’s multiple comparisons test, *n* = 4–5 per group). ns, not significant; **p* < 0.05; ***p* < 0.01; ****p* < 0.001. NT, normothermia; HT, hypothermia.

**
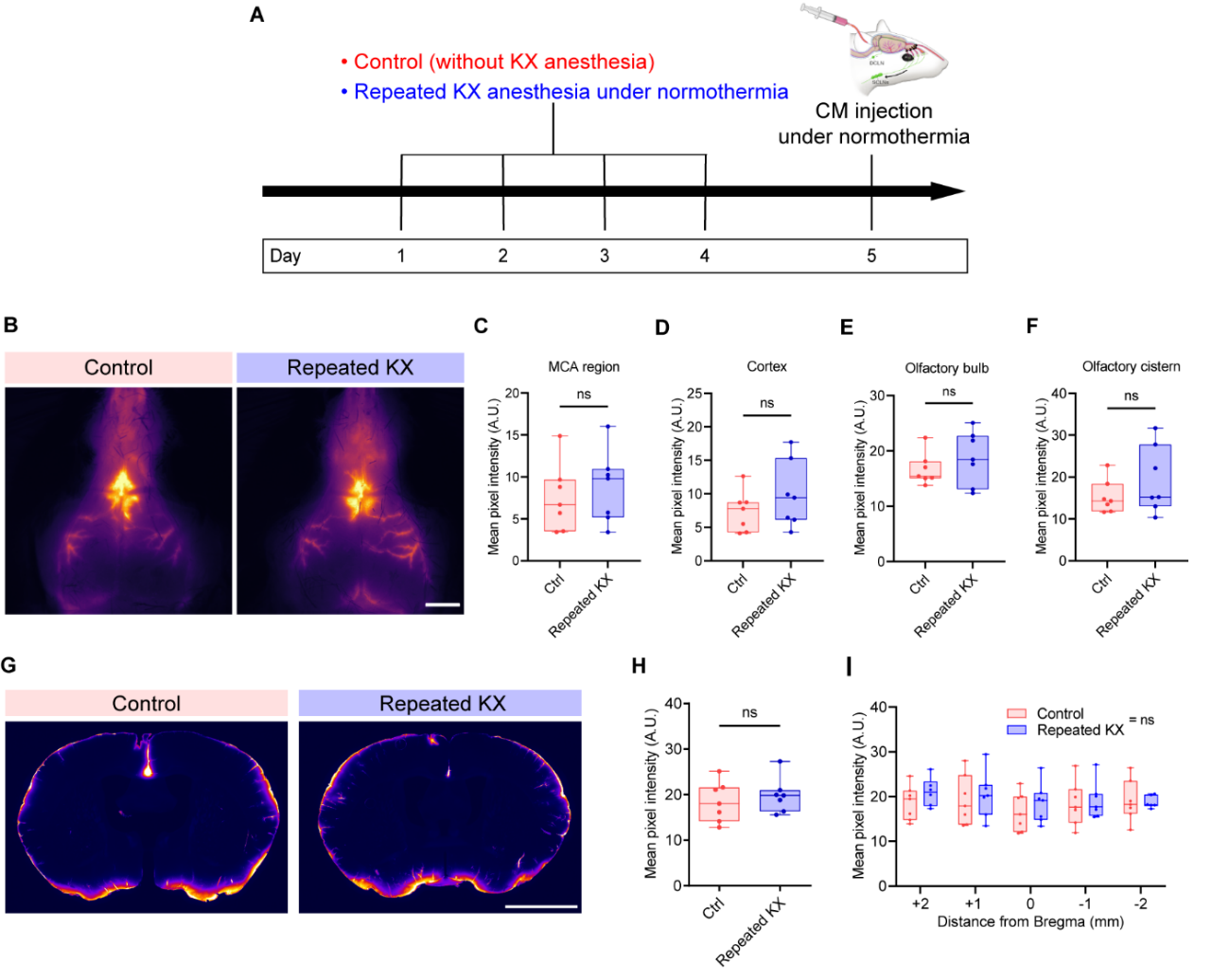
Supplementary Figure 4. Repeated anesthesia under normothermia does not impair glymphatic function. (A**) Schematic of the experiment. **(B)** Representative in vivo transcranial images from control and repeated KX anesthesia groups. Scale bar, 2.5 mm. **(C–F)** Quantitative analysis of mean tracer intensity in the MCA region, cortex, olfactory bulb, and olfactory cistern (unpaired Student’s *t*-test, *n* = 7 per group). **(G)** Representative images of coronal brain slices from control and repeated KX anesthesia groups. Scale bar, 2.5 mm. **(H)** Quantitative analysis of tracer influx in coronal brain slices averaged across five slices per brain (unpaired Student’s *t*-test, *n* = 7 per group). **(I)** Quantitative analysis of tracer influx in coronal brain slices by location (–2, –1, 0, +1, +2 mm from bregma) (two-way ANOVA with Tukey’s multiple comparisons test, *n* = 7 per group). ns, not significant. KX, ketamine/xylazine; CM, cisterna magna; MCA, middle cerebral artery.

**
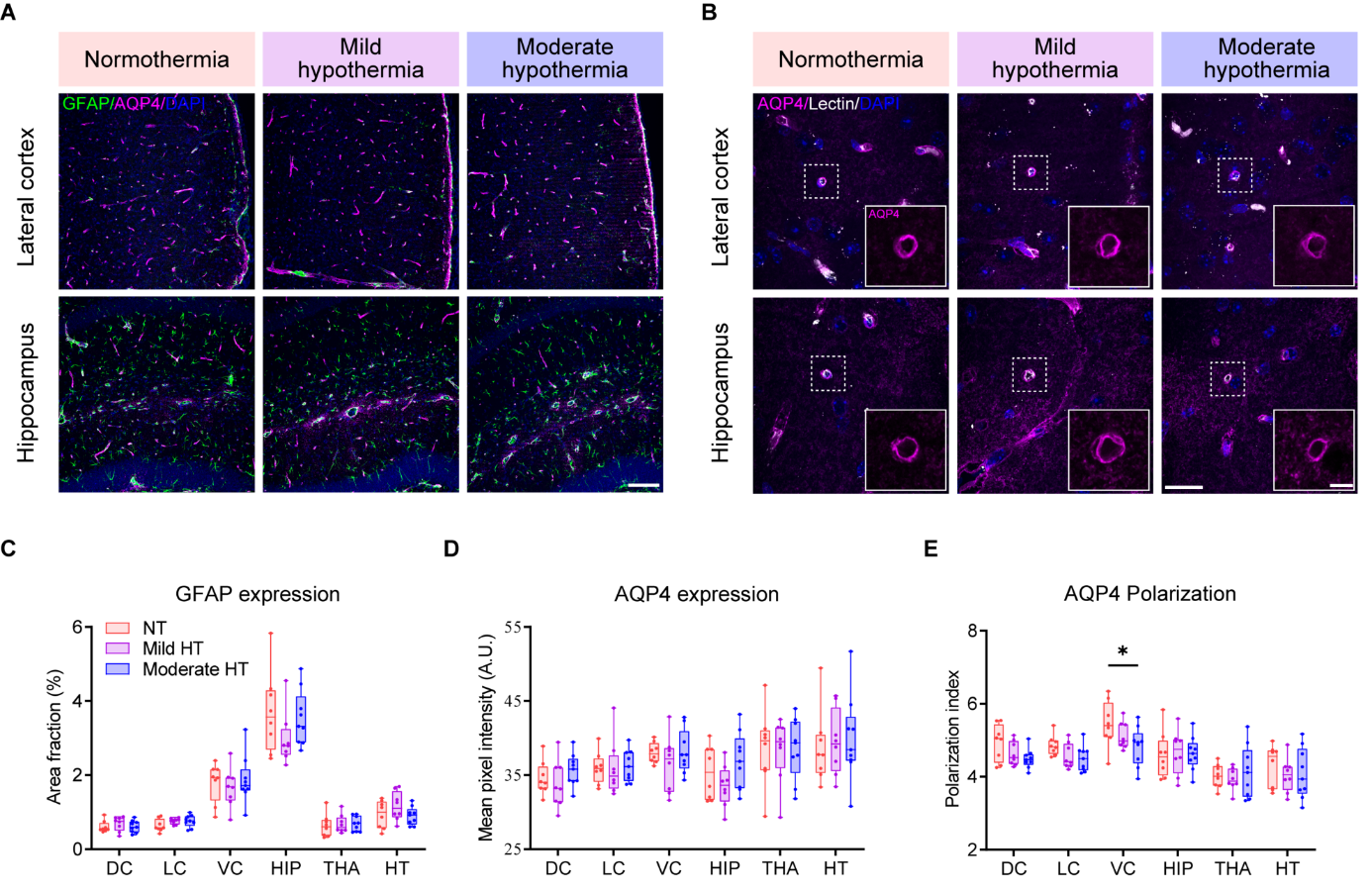
Supplementary Figure 5. GFAP and AQP4 expressions under acute hypothermia. (A)** Representative confocal images showing immunostaining of GFAP (green) and AQP4 (magenta) in the lateral cortex and hippocampus. Scale bar, 100 µm. **(B)** Representative confocal images of AQP4 (magenta) staining around lectin (white)-labelled blood vessels in the lateral cortex and hippocampus. Scale bar, 20 µm; inset, 5 µm. **(C)** Quantitative analysis of GFAP area fraction by region (two-way ANOVA with Tukey’s multiple comparisons test, *n* = 8–9 per group). **(D)** Quantitative analysis of global AQP4 mean fluorescent intensity by region (two-way ANOVA with Tukey’s multiple comparisons test, *n* = 8–9 per group). **(E)** Quantitative analysis of the AQP4 polarization index by region (two-way ANOVA with Tukey’s multiple comparisons test, *n* = 8–9 per group). **p* < 0.05. NT, normothermia; HT, hypothermia; DC, dorsal cortex; LC, lateral cortex; VC, ventral cortex; HIP, hippocampus; THA, thalamus; HT, hypothalamus; GFAP, glial fibrillary acidic protein; AQP4, aquaporin-4.

**
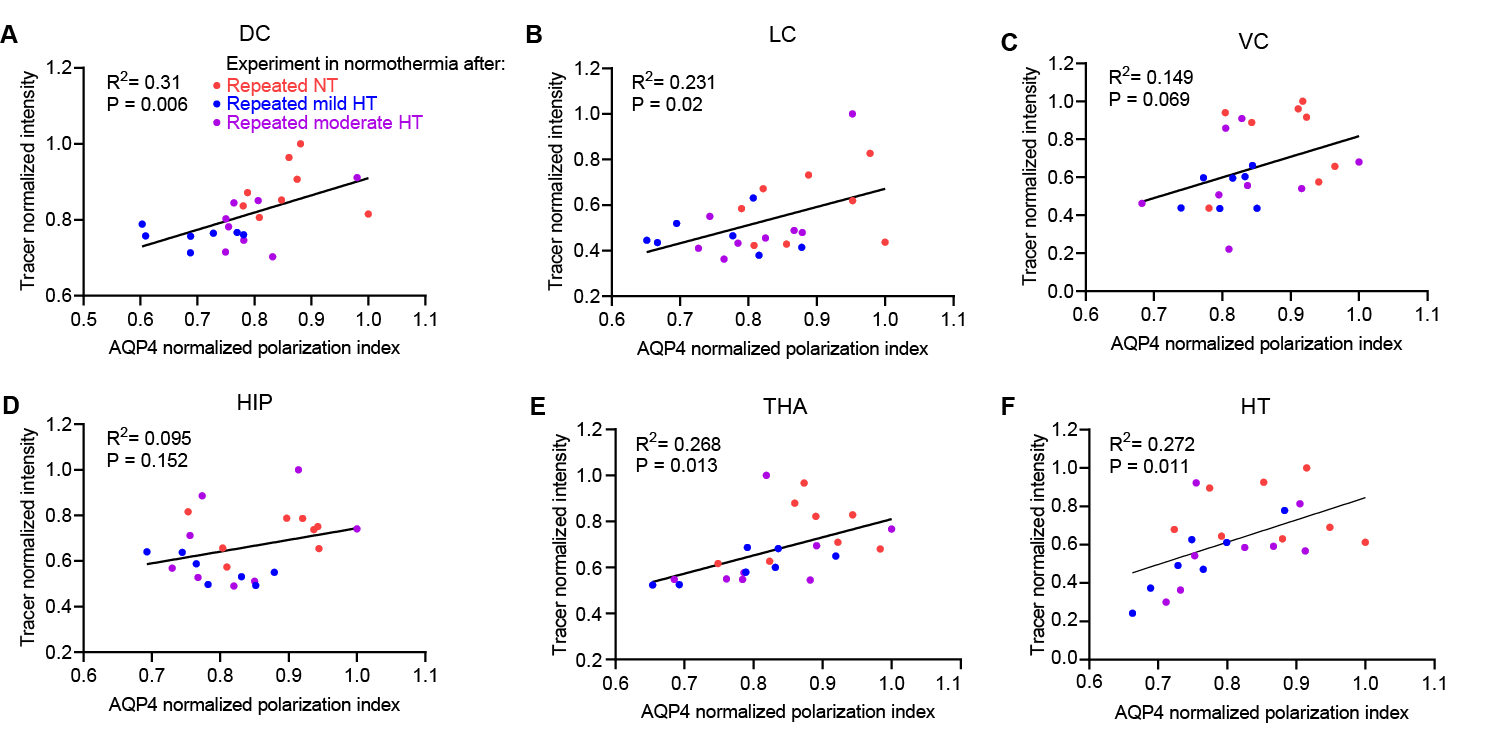
Supplementary Figure 6. AQP4 polarization correlated with tracer influx after repeated hypothermia. (A–F)** Linear regression analysis showed that AQP4 polarization was significantly associated with tracer penetration in the dorsal cortex (A), lateral cortex (B), thalamus (E), and hypothalamus (F), but not in the ventral cortex (C) or hippocampus (D) (*n* = 7–8 per group). All values were normalized to the highest value for ease of visualization and comparison. NT, normothermia; HT, hypothermia; DC, dorsal cortex; LC, lateral cortex; VC, ventral cortex; HIP, hippocampus; THA, thalamus; HT, hypothalamus; AQP4, aquaporin-4.

**Supplemental Video 1.**

Representative 3D light-sheet reconstruction of tracer distribution in a cleared brain from the normothermia group. Scale bar: 1 mm.

**Supplemental Video 2.**

Representative 3D light-sheet reconstruction of tracer distribution in a cleared brain from the mild hypothermia group. Scale bar: 1 mm.

**Supplemental Video 3.**

Representative 3D light-sheet reconstruction of tracer distribution in a cleared brain from the moderate hypothermia group. Scale bar: 1 mm.
